# Initial Raymond–Roy Occlusion Classification but not Packing Density Defines Risk for Recurrence after Aneurysm Coiling

**DOI:** 10.1007/s00062-020-00926-x

**Published:** 2020-07-01

**Authors:** Tobias Greve, Maximilian Sukopp, Maria Wostrack, Egon Burian, Claus Zimmer, Benjamin Friedrich

**Affiliations:** 1 Department of Diagnostic and Interventional Neuroradiology, Klinikum rechts der Isar, TUM School of Medicine, Technical University of Munich, Ismaninger Str. 22, Munich, 81675 Germany; 2 Department of Neurosurgery, University Hospital, LMU Munich, Marchioninistr. 15, Munich, 81377 Germany; 3 Department of Neurosurgery, Klinikum rechts der Isar, TUM School of Medicine, Technical University of Munich, Ismaninger Str. 22, 81675 Munich, Germany

**Keywords:** Intracranial aneurysm, Endovascular treatment, Aneurysm regrowth, Balloon-assisted coiling

## Abstract

**Purpose:**

After coil embolization of intracranial aneurysms, it is currently not well understood whether the initial coil packing density or the type of aneurysm residual perfusion, depicted by the modified Raymond-Roy occlusion classification, primarily effects the rate of aneurysm recurrence. We hypothesized that these factors interact and only one remains an independent risk factor.

**Methods:**

In this single center retrospective study, 440 patients with intracranial ruptured and unruptured aneurysms between 2010 and 2017 were screened. A total of 267 patients treated with stand-alone coiling, with or without stent or balloon assistance were included (age 54.1 ± 12.2 years, sex 70.4% female). Flow diverter or Woven EndoBridge (WEB) device implantation were exclusion criteria.

**Results:**

Using a binary logistic regression model, independent risk factors for aneurysm recurrence were postinterventional modified Raymond-Roy occlusion classification class (Odds ratio [OR] 1.747, 95% confidence interval [CI] 1.231–2.480) and aneurysm diameter (OR 1.145, CI 1.032–1.271). A trend towards a higher recurrence in ruptured aneurysms did not reach significance (OR 1.656, CI 0.863–3.179). Aneurysm localization, packing density, and neck width were not independently associated with aneurysm recurrence.

**Conclusion:**

Independent risk factors for aneurysm recurrence after coil embolization with and without stent or balloon assistance were aneurysm diameter and postinterventional grading within the modified Raymond-Roy occlusion classification. Packing density interacted with the latter and was not independently associated to recurrence.

## Introduction

The advent of endovascular embolization techniques for intracranial aneurysms in the last 30 years has led to continuously decreased complication and treatment failure rates. Large trials in 2003 and 2005 found similar or lower rates of morbidity and mortality compared to microsurgical aneurysm clipping [1–6]; however, patients with subarachnoid hemorrhage due to ruptured aneurysms subjected to endovascular coil embolization were found to be at higher risk for rebleeding, which in turn is mainly attributed to aneurysm recurrence [7]. Aneurysm recurrence after coil embolization is a significant risk and has been shown to occur in up to 34% of cases, with an average recurrence rate of around 20% [7–12]. It was shown that the initial packing density influences coil compaction rates which ultimately results in aneurysm recurrence [13, 14].

Beyond the aneurysm packing density, a classification system was implemented which allows characterization of residual aneurysm perfusion, named the modified Raymond–Roy occlusion classification (MRRC). It is the current gold standard for qualitative aneurysm occlusion evaluation. According to this system, an aneurysm with a class IIIb residual perfusion was shown to be associated with a higher risk of residual perfusion over time (80.8% vs. 46.4%) and a higher recanalization rate (65.1% vs. 27.4%), compared to class IIIa and II residual perfusions [15–17].

It is currently not well understood whether the initial packing density or the type of aneurysm residual perfusion primarily effects the rate of aneurysm recurrence. We hypothesize that these factors interact and thus only one remains an independent risk factor of aneurysm recurrence.

We set out to test our hypothesis in a retrospective approach on a large cohort of patients with intracranial ruptured and unruptured aneurysms that were subjected to coil embolization.

## Material and Methods

### Study Design

This was a single center retrospective study of 440 patients undergoing endovascular treatment of ruptured or unruptured saccular aneurysms between June 2010 and September 2017 were screened for eligibility. Inclusion criteria regarding treatment technique were stand-alone, stent-assisted or balloon-assisted coil embolization. The age limit was 18 years or older. Exclusion criteria were pretreated aneurysms, missing follow-up digital subtraction angiography (DSA), incomplete information about the coils used, inadequately calibrated angiography system for volume calculation and embolization techniques using Woven EndoBridge (WEB) devices (Sequent Medical, Inc, Aliso Viejo, CA, USA), flow-diverters or parent vessel occlusion. Written informed consent was waived by the local institutional review board for this study because of its retrospective character and the analyses being based only on data acquired during clinical routine.

**Fig. 1:**
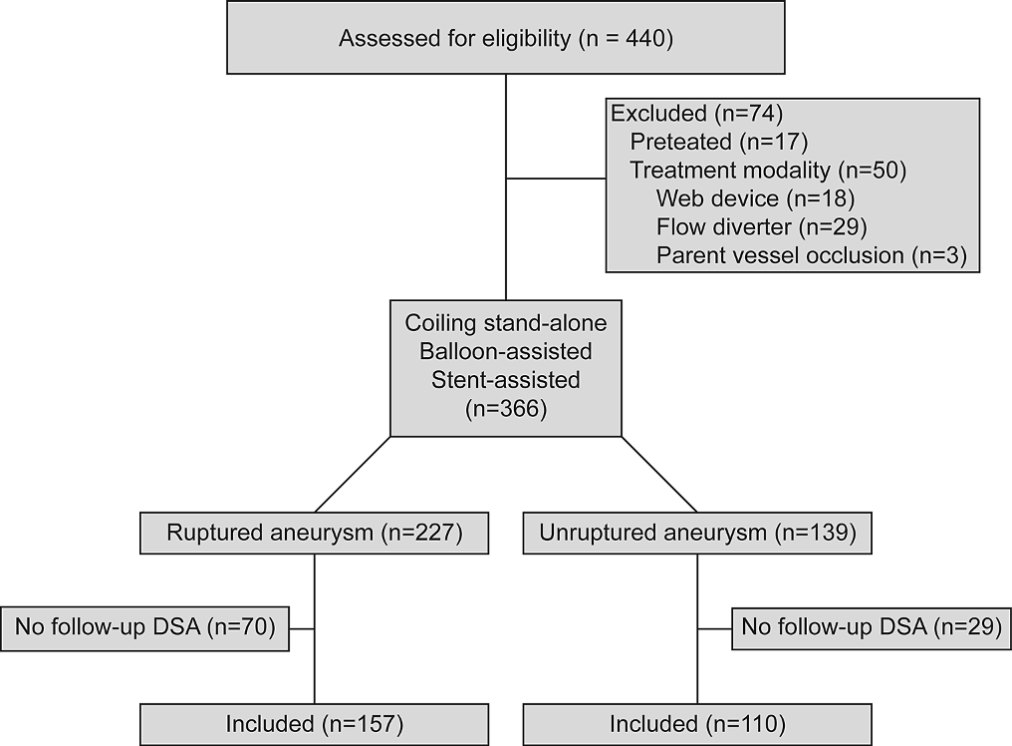
Consort scheme for patient inclusion

Of all 440 screened patients, 267 patients were included, 17 patients were excluded due to previous treatment of the aneurysm, 18 due to WEB device implantation, 29 due to flow-diverter implantation, three due to parent vessel occlusion and 99 due to missing DSA follow-up data (Fig. 1).

Of note, every patient was discharged with a scheduled follow-up DSA if clinically feasible but due to the supraregional nature of the treatment center, a significant proportion were followed-up at their proximate home institution. Furthermore, patients with low clinical performance status after subarachnoid hemorrhage were frequently followed-up by computed tomography (CT) angiography only.

### Endovascular Treatment

An indication for endovascular intervention versus surgical clipping was established based on consensus in our institutional vascular board consisting of experienced interventional neuroradiologists and neurosurgeons. Endovascular coil embolization was performed with the patient under general anesthesia and with systemic administration of heparin. The choice of which coil type to use was mainly influenced by aneurysm characteristics, such as volume, neck width, and maximum diameter (Table 1). The aim of coil placement was defined as packing aneurysms as densely as possible, with coils being incorporated into the aneurysm sac until no more material could be inserted.

**Table 1:** Material

Device	Manufacturer
*Orbit Galaxy, Trufill*	Codman & Shurtleff, Inc., Raynham, MA, USA
*HyperSoft 3D, HydroCoil, Cosmos, Complex, Compass*	Microvention, Inc., Aliso Viejo, CA, USA
*Axium*	Covidien, Dublin, Irland
*Target, GDC*	Stryker, Fremont, CA, USA
*Diameter range 0.0095–0.015 inch, PC400*	Penumbra, Inc., Alameda, CA, USA

### Analysis and Outcome Definitions

Aneurysma characteristics were analyzed by reviewing angiographic data. Aneurysm volume and packing density were calculated using the AngioSuite system (Cascade Medical, Knoxville, TN, USA). The calculation of the packing density is based on aneurysm volume and on the number and type of coils employed and was previously shown to be highly accurate [18].

Aneurysms were divided into diameter categories proposed by the authors of the International Study of Unruptured Intracranial Aneurysms: small <7 mm, medium 7–12 mm, large >12–25 mm [2]. Dome-to-neck ratio was defined as the dome diameter divided by the aneurysm neck. To determine procedure success; we evaluated grade of occlusion by applying the MRRC classification after the initial procedure and on follow-up DSA imaging [16]. Follow-up angiograms were further dichotomized into present or absent coil compaction, which was defined as shrinkage of the coil mesh or increased contrast agent filling within the coil interstices compared with the postinterventional angiogram. Aneurysm recurrence was defined as a significant new aneurysm neck or dome filling; which required retreatment of the aneurysm. Aneurysm recurrence was in most cases (but not exclusively) represented by a deterioration of an MRRC classes I to II, IIIa or IIIb; or from an MRRC class II to IIIa or IIIb. Procedure time was defined as the time between first image acquisition and the first control image.

### Data Analyses and Statistics

Analysis was performed on all included aneurysms. Multiple aneurysms seen in the same patient were treated as independent observations. Continuous variables with a normal distribution were compared using Welch’s *t*-test for independent samples. The Mann–Whitney *U*-test was used for non-normally distributed data. Categorical variables and frequency counts were compared using Fisher’s exact test. All noted *p* values are two sided and *p* < 0.05 is considered significant, *p *< 0.01 highly significant and *p* < 0.001 very highly significant. If not otherwise specified, normally distributed variables are shown as mean ± standard deviation. Non-normally distributed data are reported as median and interquartile range. Variables which showed significant association with aneurysm recurrence were fitted into a binary logistic regression model. If the variables showed significant multicollinearity, they were not included in the model. Data management and statistical analyses were performed using SPSS statistics V26.0 (IBM Corporation, Armonk, NY, USA).

## Results

### Patient Demographics and Aneurysm Properties

The average age was 54.1 ± 12.2 years. The cohort consisted of 188 (70.4%) female patients. A ruptured intracranial aneurysm was treated in 157 (58.8%) patients (RIA group), and an unruptured intracranial aneurysm was treated in 110 (41.2%) patients (UIA group). Between the RIA and UIA groups, the distribution of aneurysm location differed significantly, with an increased number of ruptured aneurysms within the anterior communicating, the posterior communicating and the vertebral arteries (*p* < 0.001). The dome-to-neck ratio was slightly larger in the RIA group (2.1 vs. 1.9, *p* = 0.024). All other demographic properties and aneurysm properties were similar between both groups (Table 2).

**Table 2:** Patient and aneurysm properties

	Ruptured aneurysm (*n* = 157, 58.8%)	Unruptured aneurysm (*n* = 110, 41.2%)	*p*-Value
Age (years)	56 ± 12	54 ± 12	0.135
Gender (female)	109 (69.4%)	79 (71.8%)	0.686
Location			
*ICA*	10 (6.4%)	23 (20.9%)	**<0.001**
*PCOM*	34 (21.7%)	18 (16.4%)	
*MCA*	9 (5.7%)	12 (10.9%)	
*ACOM*	74 (47.1%)	27 (24.5%)	
*ACA*	6 (3.8%)	9 (8.2%)	
*BA*	19 (12.1%)	18 (16.4%)	
*VA*	5 (3.2%)	1 (0.9%)	
*PCA*	0 (0%)	2 (1.8%)	
Aneurysm maximum diameter (mm)	6.9 ± 3.3	7.1 ± 4.3	0.706
Aneurysm neck diameter (mm)	3.0 ± 2.5	3.3 ± 1.8	0.409
Dome to neck ratio	2.1 ± 0.9	1.9 ± 0.7	**0.024**
Aneurysm size groups			
*<7* *mm*	89 (56.7%)	62 (56.4%)	0.378
*7–12* *mm*	58 (36.9%)	36 (32.7%)	
*>12* *mm*	10 (6.4%)	12 (10.9%)	
Aneurysm volume (mm^3^)	152 ± 447	223 ± 493	0.237
Aneurysm volume groups			
*≤15* *mm*^*3*^	29 (18.5%)	26 (23.6%)	0.702
*>15–35* *mm*^*3*^	33 (21.0%)	20 (18.2%)	
*>35–75* *mm*^*3*^	31 (19.7%)	18 (16.4%)	
*>75–200* *mm*^*3*^	37 (23.6%)	23 (20.9%)	
*>200* *mm*^*3*^	27 (17.2%)	23 (20.9%)	

Gender, Location, Aneurysm size groups, Aneurysm volume groups are presented as absolute counts and percent. Age, aneurysm diameter, aneurysm neck diameter, dome-to-neck ratio and aneurysm volume are presented as mean ± standard deviation. Emphasized values (bold) indicate statistical significance. *p* < 0.05 is significant, *p* < 0.01 is highly significant, *p* < 0.001 is very highly significant

*ICA* internal carotid artery, *PCOM* posterior communicating artery, *MCA* medial cerebral artery, *ACOM* anterior communicating artery, *ACA* anterior cerebral artery, *BA* basilar artery, *VA* vertebral artery, *PCA* posterior cerebral artery

### Procedure Characteristics

Most procedures consisted of coiling without balloon or stent-assisted techniques (67.6%, Table 3); however, coiling without balloon or stent assistance was more frequently employed in the RIA group (79.0%) than in the UIA group (48.2%, *p* < 0.001). Balloon-assisted and stent-assisted techniques or a combination of both were more frequently applied in the UIA group compared to the RIA group (*p* < 0.001). The average procedure time, median number of coils used, average packing density and proportions of postprocedural MRRC classes were not significantly different between the RIA and UIA groups (*p* > 0.05), with most patients showing no aneurysm rest after coil embolization, representing an MRRC class I occlusion (49.7% vs. 46.4%). The most frequent procedure-related complication was an arterioarterial thrombotic event during embolization (13.4% in the RIA and 7.3% in the UIA group, *p* > 0.05), while most patients did not exhibit any procedure-related complications (68.2% RIA vs. 79.1% UIA group, *p* > 0.05).

**Table 3:** Procedure and follow-up

	Ruptured aneurysm (*n* = 157, 58.8%)	Unruptured aneurysm (*n* = 110, 41.2%)	*p*-Value
Procedure time (min)	123 ± 59	139 ± 77	0.080
Technique			
*Simple coiling*	124 (79.0%)	53 (48.2%)	**<0.001**
*Balloon-assisted coiling*	21 (13.4%)	22 (20.0%)	
*Stent-assisted coiling*	10 (6.4%)	32 (29.1%)	
*Balloon and stent-assisted coiling*	2 (1.3%)	3 (2.7%)	
Number of coils	3 [IQR 2–5]	3 [IQR 2–5]	0.903
Packing density (%)	37.0 ± 15.0	36.8 ± 17.2	0.893
MRRC class after procedure			
*I*	78 (49.7%)	51 (46.4%)	0.080
*II*	41 (26.1%)	19 (17.3%)	
*IIIa*	27 (17.2%)	24 (21.8%)	
*IIIb*	11 (7.0%)	16 (14.5%)	
Procedure-related events			
*None*	107 (68.2%)	87 (79.1%)	0.226
*Thromboembolic event*	9 (5.7%)	8 (7.3%)	
*Rupture*	4 (2.5%)	2 (1.8%)	
Time to follow-up (months)	7.3 ± 4.0	8.6 ± 4.4	**0.011**
MRRC class at follow-up			
*I*	73 (46.5%)	71 (64.5%)	**0.022**
*II*	47 (29.9%)	20 (18.2%)	
*IIIa*	8 (5.1%)	2 (1.8%)	
*IIIb*	29 (18.5%)	17 (15.5%)	
Coil compaction at follow-up	60 (38.2%)	26 (23.6%)	**0.016**
Aneurysm recurrence at follow-up	40 (25.5%)	22 (20.0%)	0.297
Modality of retreatment			
*Coiling*	32 (80.0%)	21 (95.5%)	0,205
*Clipping*	8 (20.0%)	1 (4.5%)	

Coil compaction and aneurysm recurrence are presented as absolute counts and percentage. Number of coils are presented as median ± IQR (interquartile range). Procedure time, packing density and time to follow-up are presented as mean ± standard deviation. Emphasized values (bold) indicate statistical significance. *p* < 0.05 is significant, *p* < 0.01 is highly significant, *p* < 0.001 is very highly significant

*MRRC* Modified Raymond–Roy occlusion classification

### Follow-up Analysis

The median time to follow-up was slightly shorter in the RIA group (7.3 months), compared to the UIA group (8.6 months, *p* = 0.011). While the distribution of postinterventional MRRC classes was not significantly different between both groups, the MRRC classes significantly differed at first follow-up (Table 3). The RIA group showed a higher number of reperfusions at first follow-up, indicated by a lower proportion of MRRC class I (46.5% vs. 64.5% for RIA vs. UIA groups, respectively, *p* = 0.022).

Aneurysms in which MRRC class I was achieved after coiling less frequently converted to MRRC classes IIIa or IIIb at first follow-up, compared to aneurysms that were classified MRRC class II after coiling (6.2% vs. 23.3%, *p* < 0.001). Aneurysms in which MRRC class IIIa was achieved after coiling tended to convert more frequently to MRRC classes I or II at first follow-up, compared to aneurysms, in which MRRC class IIIb was achieved (62.7% vs. 44.4%, *p* = 0.123).

Coil compaction was more frequent in the RIA group (38.0% vs. 23.6%, *p* = 0.016). The overall rate of aneurysm recurrence was 23.2% with a higher recurrence rate in the RIA group, compared to the UIA group (25.5% vs. 20.0%, *p* = 0.297).

### Risk Factors for Aneurysm Recurrence

We further investigated factors influencing aneurysm recurrence. A highly significant difference in aneurysm diameter between recurrent and non-recurrent aneurysms was observed (8.8 ± 5.0 mm vs. 6.4 ± 3.0 mm, *p* < 0.001). Similarly, aneurysm neck width (3.6 ± 2.1 mm vs. 3.0 ± 2.2 mm, *p* < 0.001) and aneurysm volume (388 ± 863 mm^3^ vs. 111 ± 183 mm^3^, *p* < 0.001) were significantly higher in recurrent aneurysms. The MRRC class was highly differently distributed among recurrent and non-recurrent aneurysms (*p* < 0.001) with 24.2% completely occluded aneurysms (MRRC class I) in the recurrent, and 55.6% completely occluded aneurysms in non-recurrent group. The relative proportion of postinterventional MRRC class IIIa and class IIIb aneurysms was almost double among all recurring aneurysms.

Initial packing density was slightly lower in recurrent aneurysms (33.0 ± 14.0% vs. 39.1 ± 20.4%, *p* = 0.018). The median number of coils deployed was significantly different between both groups (4, interquartile range 2.0–6.25 vs. 3, interquartile range 2.0–5, *p* = 0.002). Localization was statistically differently distributed with a higher representation of internal cerebral artery, anterior cerebral artery and vertebral artery aneurysms and an underrepresentation of posterior communicating artery, medial cerebral artery and anterior communicating artery aneurysms among all recurrent aneurysms (*p* = 0.048).

Significant correlation was observed between aneurysm diameter and aneurysm volume (r = 0.766, *p* < 0.001) as well as number of coils employed (r = 0.720, *p* < 0.001). Medium correlation was observed between aneurysm diameter and aneurysm neck width (r = 0.519, *p* < 0.001). In addition, aneurysm volume significantly correlated to the number of coils employed (r = 0.607, *p* < 0.001).

No significant differences between recurrent and non-recurrent aneurysms were observed for age, gender, dome-neck ratio, procedure time, time to follow-up, rupture status, technique (coiling alone versus stent-assisted coiling versus balloon-assisted coiling or a combination of those), complications, coil type, coil manufacturer and the interventionalist.

### Independent Risk Factors for Aneurysm Recurrence

We fitted all variables significantly associated to aneurysm recurrence in the previous univariate analysis into a binary logistic regression model. We excluded aneurysm volume and the number of coils deployed due to their significant collinearity to aneurysm diameter and we included rupture status due to significant differences in MRRC classes at first follow-up after coiling of ruptured vs. unruptured aneurysms.

**Fig. 2:**
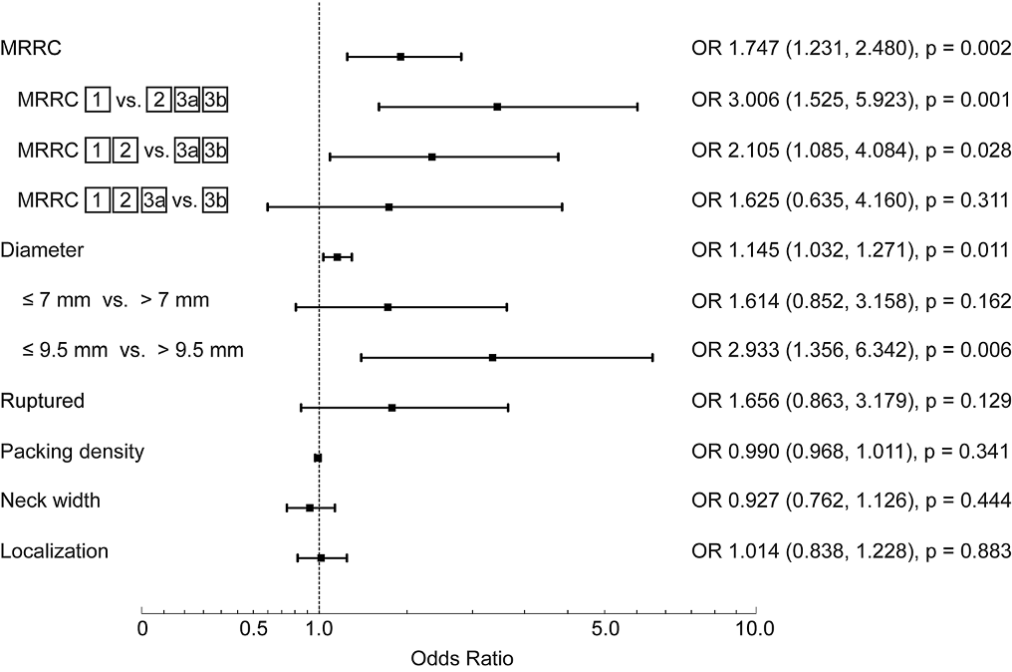
Independent risk factors for aneurysm recurrence. Values in parentheses represent lower and upper boundaries of the 95% confidence interval. *p* < 0.05 is significant, *p* < 0.01 is highly significant, *p* < 0.001 is very highly significant. *MRRC* modified Raymond–Roy occlusion classification, *OR* odds ratio

In this model, the only independent risk factors for aneurysm recurrence were found to be postinterventional MRRC class (Odds ratio [OR] 1.747, 95% confidence interval [CI] 1.231–2.480) and aneurysm diameter (OR 1.145, CI 1.032–1.271). We observed a trend towards a higher recurrence in ruptured aneurysms, without reaching statistical significance (OR 1.656, CI 0.863–3.179). Aneurysm localization, packing density, and neck width were not independently associated with aneurysm recurrence (Fig. 2).

To further investigate the influence of MRRC classes on aneurysm recurrence, the MRRC class was dichotomized and the binary logistic regression model was repeated (Fig. 2). We observed the highest value of increased odds when MRRC class was dichotomized between class I vs. II–IIIb (OR 3.006, CI 1.525–5.923). When dichotomizing between MRRC classes I–II versus IIIa–IIIb, the OR was 2.105 (CI 1.085–4.084). Dichotomizing between MRRC class I–IIIa vs. IIIb did not yield a significant increase in odds of recurrence (OR 1.625, CI 0.635–4.160).

We further aimed to analyze the odds for aneurysm recurrence if aneurysms exceeded certain diameter thresholds (Fig. 2). For that aim, we dichotomized aneurysm diameter in ≤7 mm and >7 mm and repeated the binary logistic regression model. The odds ratio for distinction was not significant (OR 1.614, CI 0.852–3.158). To find a significant diameter threshold we performed a ROC analysis and determined the best cut-off between sensitivity and specificity using the Youden’s index. With this method, a diameter threshold of 9.5 mm was determined to yield the best trade-off between both parameters. The binary logistic regression model was repeated using dichotomized groups with a diameter ≤9.5 mm versus a diameter >9.5 mm. With this distinction, the OR for recurrence was significant (OR 2.933, CI 1.356–6.342).

## Discussion

We were able to show on this retrospective cohort of ruptured and unruptured intracranial aneurysms that independent risk factors for aneurysm recurrence after coil embolization are aneurysm diameter and postinterventional MRRC class. We further showed that achieving postinterventional MRRC class I (over II to IIIb) or II (over IIIa/IIIb) is crucial since this significantly reduces the odds of recurrence. Furthermore, aneurysms with a diameter >9.5 mm manifest an almost three times higher risk for recurrence compared to smaller aneurysms.

Comparing our data to the literature, typical demographic parameters, e.g. age, sex distribution and aneurysm diameter distribution were comparable to published data [6, 16]. Furthermore, procedure times, complete occlusion rate (MRRC class I) after embolization as well as MRRC classes at first follow-up were comparable to published data [10, 16]. Overall aneurysm recurrence ranged between 25.5% in the RIA and 20.0% in the UIA group, which is also comparable to previous literature [10, 19].

In previous studies, several factors have been identified to be associated to recurrence of endovascularly treated aneurysms. These comprise aneurysm dome and neck diameter [10, 11, 20], rupture status [10, 21, 22] and degree of occlusion [10, 16, 23–26]. There are mixed reports on influence of packing density on recurrence rates [9, 13, 27], with one large trial in particular not reporting a relationship between aneurysm recurrence and packing density [28]. Similarly, mixed reports exist on the influence of aneurysm localization with most studies not reporting an association [10, 21, 29].

In our series, aneurysm size was an independent risk factor for recurrence. Interestingly, surpassing a diameter threshold of 7 mm did not significantly increase the odds for recurrence while the odds were almost three times as high when aneurysms surpassed 9.5 mm in diameter. This is an important finding since it enables the clinician to schedule follow-up imaging studies at an earlier time point in those patients at risk.

Although aneurysm neck width was significantly associated with aneurysm recurrence in univariate analysis, it failed to remain an independent risk factor in the multivariate analysis, most likely due to high collinearity with other factors, mainly aneurysm dome diameter.

Rupture status was associated with a higher risk for recurrence in our series (25.5% vs. 20.0%) but it failed to remain an independent factor in multivariate analysis. Since our study was retrospective and patient populations and techniques were heterogeneous, we cannot claim that this finding stands in contrast to previously published data, but we postulate that there are more important factors to consider when evaluating risk of aneurysm recurrence.

In agreement with previous literature [10, 16, 23–26], we found the degree of aneurysm occlusion to be significantly associated with reduced aneurysm recurrence rate. The original Raymond–Roy occlusion classification encompasses classes I (complete occlusion), II (residual neck) and III (residual aneurysm) [10]. Although it was not designed to predict aneurysm recurrence, studies showed higher recurrence rates in class III aneurysms [30, 31]. Mascitelli et al. appended this classification by subdividing class III into classes IIIa (contrast filling of coil interstices) and IIIb (contrast filling outside the coil interstices along the aneurysm wall) [16]. The authors reported a significantly higher conversion of class IIIa aneurysms to classes I and II (83%) compared to class IIIb aneurysms into classes I and II (15%).

In our data, the conversion rate from IIIa to I or II was 62.7% and from IIIb to I or II was 44.4%, which was not significantly different. This somewhat less pronounced difference in favorable outcome between classes IIIa and IIIb also translated into OR of aneurysm recurrence: We report the highest increase in OR for recurrence if no complete occlusion (MRRC classes II, IIIa, IIIb) is achieved compared to complete occlusion (MRRC class I). A still significantly higher OR in favor of recurrence is observed if incomplete occlusion with MRRC classes IIIa or IIIb is achieved in opposition to classes I and II (Fig. 2); however, differentiating between class IIIb versus classes I, II and IIIa did not significantly change the odds of recurrence.

The packing density was not significantly different between the RIA and UIA group in our series although it is widely regarded safer to not try to maximize packing attenuation in ruptured aneurysms. Although packing density tended to be lower in recurring aneurysms, it was not shown to be an independent risk factor in multivariate analysis. Of note, authors previously suspected that increasing packing density beyond a threshold of 20% does not necessarily further reduce recurrence rate [32]. Since the average packing density in our study was relatively high (38%), this might be one explanation for the loose association between packing density and aneurysm recurrence rate in this cohort. The mixed findings on this topic in previous studies corroborate our data [9, 27, 28].

While studies about the association of aneurysm localization to recurrence have shown mixed results [10, 21, 29], we saw a mild association of those parameters in our series, with a higher representation of the internal cerebral artery, anterior cerebral artery and vertebral artery and an underrepresentation of the medial cerebral artery, posterior communicating artery and anterior communicating artery among recurring aneurysms; however, localization did not prove to be an independent risk factor in multivariate analysis.

A major limitation of this study is the retrospective character. Second, data on rebleeding rates were not available as part of our study since patients were often treated for rebleeding in other hospitals. Third, the extended period of inclusion between the years 2010 and 2017 encompassed significant technical developments in endovascular aneurysm treatment. Although we aimed to counteract this caveat by excluding aneurysm treatment with WEB devices and flow diverters, it remains a major limitation. Finally, a significant proportion of patients were lost to follow-up. On one hand, due to the supraregional aspect of the hospital, patients were often followed up at their proximate home institution. Furthermore, patients with a low clinical performance status after subarachnoid hemorrhage frequently only received CT angiography follow-up.

## Conclusion

In this retrospective study on endovascular treatment of ruptured and unruptured intracranial aneurysms, independent risk factors for aneurysm recurrence after coil embolization with and without stent or balloon assistance were aneurysm diameter and postinterventional MRRC class. We confirmed that achieving postinterventional MRRC class I (over II, IIIa and IIIb) or II (over IIIa and IIIb) but not IIIa significantly decreases the odds of recurrence. The MRRC class independently influences risk of recurrence and stands in contrast to packing density, which failed to remain an independent predictor of recurrence.

In contrast to previous literature, a critical aneurysm diameter threshold of >9.5 mm was found, since these aneurysms show a three times higher risk for recurrence.

## Abbreviations

CI: 95% Confidence interval
CT: Computed tomography
DSA: Digital subtraction angiography
MRRC: Modified Raymond–Roy occlusion classification
OR: Odds ratio
RIA: Ruptured intracranial aneurysm
UIA: Unruptured intracranial aneurysm
WEB: Woven EndoBridge Device
